# Spatiotemporal prediction of vancomycin-resistant Enterococcus colonisation

**DOI:** 10.1186/s12879-022-07043-9

**Published:** 2022-01-20

**Authors:** J. M. van Niekerk, M. Lokate, L. M. A. Braakman-Jansen, J. E. W. C. van Gemert-Pijnen, A. Stein

**Affiliations:** 1 Department of Psychology, Health and Technology/Center for eHealth Research and Disease Management, Faculty of Behavioural Sciences, University of Twente, Enschede, The Netherlands; 2 Department of Earth Observation Science, Faculty of Geo-Information Science and Earth Observation (ITC), University of Twente, Enschede, The Netherlands; 3 Department of Medical Microbiology, University of Groningen, University Medical Center Groningen, Groningen, The Netherlands

**Keywords:** Vancomycin-resistant enterococci, Intrahospital patient movements, Spatiotemporal risk factors, Dynamic directed spatiotemporal graph, Centrality measure, Healthcare decision support

## Abstract

**Background:**

Vancomycin-resistant enterococci (VRE) is the cause of severe patient health and monetary burdens. Antibiotic use is a confounding effect to predict VRE in patients, but the antibiotic use of patients who may have frequented the same ward as the patient in question is often neglected. This study investigates how patient movements between hospital wards and their antibiotic use can explain the colonisation of patients with VRE.

**Methods:**

Intrahospital patient movements, antibiotic use and PCR screening data were used from a hospital in the Netherlands. The PageRank algorithm was used to calculate two daily centrality measures based on the spatiotemporal graph to summarise the flow of patients and antibiotics at the ward level. A decision tree model was used to determine a simple set of rules to estimate the daily probability of patient VRE colonisation for each hospital ward. The model performance was improved using a random forest model and compared using 30% test sample.

**Results:**

Centrality covariates summarising the flow of patients and their antibiotic use between hospital wards can be used to predict the daily colonisation of VRE at the hospital ward level. The decision tree model produced a simple set of rules that can be used to determine the daily probability of patient VRE colonisation for each hospital ward. An acceptable area under the ROC curve (AUC) of 0.755 was achieved using the decision tree model and an excellent AUC of 0.883 by the random forest model on the test set. These results confirms that the random forest model performs better than a single decision tree for all levels of model sensitivity and specificity on data not used to estimate the models.

**Conclusion:**

This study showed how the movements of patients inside hospitals and their use of antibiotics could predict the colonisation of patients with VRE at the ward level. Two daily centrality measures were proposed to summarise the flow of patients and antibiotics at the ward level. An early warning system for VRE can be developed to test and further develop infection prevention plans and outbreak strategies using these results.

## Background

Vancomycin-resistant enterococci (VRE) was first reported in Europe in 1986 [1] and since then has been the cause of severe health and monetary burdens [2]. The prevalence of VRE and VRE outbreaks have increased over the past 20 years in Europe [3]. *Enterococcus faecalis* and *Enterococcus faecium* are the Enterococci species typically found in humans’ gastrointestinal tracts, which could lead to bacteraemia, endocarditis, intra-abdominal and pelvic infections and urinary tract infections [1]. Patients are more than twice as likely to die from bloodstream infections caused by VRE as compared to a susceptible strain of Enterococcus [4]. Enterococci have properties that make them naturally resistant to the most used antimicrobial, and in particular, they can quickly become resistant to any new last-resort antimicrobials introduced.

Enterococci can survive on hospital surfaces and spread between patients and healthcare workers (HCW) using hands and surfaces as vectors [5]. In addition to direct patient-patient and HCW-HCW transmission pathways, there are five main transmission pathways for VRE inside a hospital: (1) patient to HCW; (2) patient to the environment; (3) HCW to patient; (4) environment to patient; (5) environment to HCW [6]. Since the VRE can survive on dry environmental surfaces for months, it could be a constant source for new outbreaks [7]. These reservoirs may persist despite routine cleaning procedures [8].

The immediate surroundings of a patient with VRE are likely to contain VRE reservoirs [9] and the odds of a patient being colonised with VRE increase when prior room occupants had VRE [10, 11]. The risk of colonisation increases as the number and proportion of patients with VRE in the same unit increases [12]. Patients also face increased odds of VRE colonisation the more days they spend hospitalised [13]. Antibiotic use and immunosuppressing comorbidities such as leukaemia have been identified as risk factors for VRE colonisation [4, 13].

When a VRE outbreak occurs in a hospital, colonised patients are isolated, the extent of the outbreak is estimated and additional control measures are implemented if necessary [3]. Estimating the extent of an outbreak involves determining the contact group, usually at the ward level. The contact group consists of the patients who could potentially have been colonised during the outbreak. Contact tracing is typically used to determine the patients at risk. To verify which patients were indeed colonised, a screening process can be carried out, which can be expensive an uncomfortable for patients [14]. The benefits of improving the estimation accuracy of these contact groups are: (1) control measures are more effective, which translates into fewer transmissions and ultimately less infections; (2) fewer patients are burdened by the screening process; (3) less testing reduces the financial burden.

Even though estimation of the extent of an outbreak plays a critical role in outbreak management, few studies have investigated the relationship between the patient movements between hospital wards and the spread of microorganisms. Reasons for patients to move from one department to the other include deterioration of health; surgery after which they are moved to intensive care and afterwards to general care or more specialised care department; hospital logistics due to limited capacity. One study used centrality measures of intrahospital patient movements to predict the onset of *clostridium difficile* at the ward level [15]. The centrality of hospital antibiotic use, however, was not considered. *Clostridium difficile* can survive on hospital surfaces and patients are at risk from environmental vectors. Recent studies have shown that each intrahospital transfer increases a patient’s odds of contracting *clostridium difficile* by 7% (95% CI 1.02–1.13). To our knowledge, no similar studies exist for the VRE.

The effects of intrahospital patient movements and antibiotic usage in hospitals are usually studied separately in antimicrobial resistance (AMR) research. The use of antibiotics is usually included as a possible confounding effect to predict VRE colonisation in patients, but the use of antibiotics of other patients who may have frequented the same ward as the patient in question is often neglected. Hospitals are dynamic systems with many moving objects and each of those objects has a surface that can act as a vector for VRE. Furthermore, antibiotic use can increase the number of VRE in patients due to selection pressure which can then spread between patients [8, 16]. For these reasons, VRE should be studied using covariates which include spatiotemporal movements of patients and antibiotics in the hospital.

This study investigates how patient movements between hospital wards and their antibiotic use can explain the colonisation of patients with VRE. We estimate the probability of a patient being colonised with VRE at the ward level using intrahospital movement data and antibiotic usage data. We estimate this probability using a decision tree model and a random forest ensemble model and compare the model performance as a sub-objective. This study is important because it allows infection prevention and control specialists and outbreak management staff to determine which wards are at risk of a VRE outbreak using commonly available hospital data.

## Methods

### Patient movement and antibiotic data

We used retrospective patient movement data from the University Medical Center Groningen (UMCG), one of the largest hospitals in the Netherlands with more than 10,000 employees and almost 1400 beds. Antibiotic usage and patient movement data are stored in an electronic health record (EHR) database. The period under study is January 2018 until December 2019. The anonymised data consist of admission and discharge dates for each department within the hospital and antibiotic administration times during admission. These data were used to calculate two covariates for each day during the period of study: (1) the number of patients in each ward (pat_num); (2) the number of patients using antibiotics in each ward (pat_num_ant).

### Spatiotemporal graph

The intrahospital patient movements data can be used to construct a dynamic directed spatiotemporal graph (DG) [17]. The graph nodes are the wards and the edges between the nodes are the patients moving between the wards. The DG is spatiotemporal and dynamic since it presents the location of patients using a node structure over time. We created two DGs using the patient movement data and the antibiotics data. The first graph includes all patient movement between all wards. The second graph only includes the movements of patients using antibiotics.

### PageRank algorithm

The PageRank (PR) algorithm aims to determine the centrality or “importance” of nodes given the number of other “important” nodes with vectors directed towards it [18]. In the context of this study, the PR algorithm estimates the probability distribution of an arbitrary patient ending up in a particular ward. We calculated the daily PageRank probabilities for both DGs using a 30-day rolling time window: (1) PageRank of patient movements between wards (PR_pat_num) and (2) PageRank of patient movements currently using antibiotics (PR_pat_num_ant). The PR_pat_num and PR_pat_num_ant represent the centrality of wards in terms of patients and antibiotics, respectively.

### VRE screening data

The number of VRE tests fluctuated between 100 to 300 per week during the study period. A polymerase chain reaction (PCR) test was performed on rectal swabs from patients. If the PCR was suspected for VRE, culturing was performed. A patient was considered to be colonised with VRE if VRE could be isolated from culturing. Next Generation Sequencing (NGS) was performed on the VRE isolates and a minimum spanning tree based on the cgMLST was used to analyse the NGS sequence data for molecular epidemiological investigation.

**Fig. 1:**
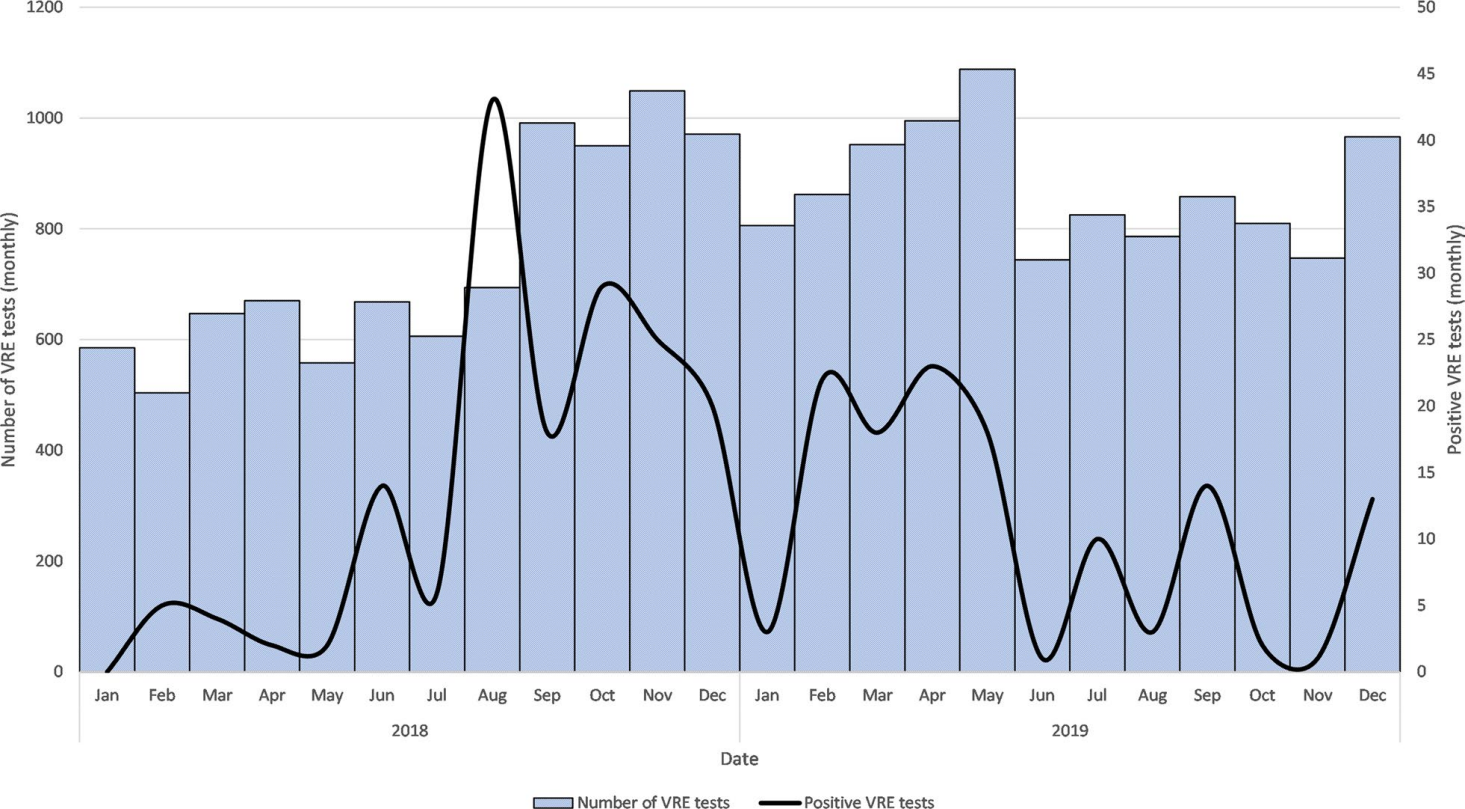
VRE tests and the number of positive VRE test results during 2018–2019

All patients directly transferring from other hospitals were tested for VRE on admission over the entire study period. In addition, intensive care units (ICU) were screened for VRE twice a week. A VRE outbreak occurred at UMCG during the second half of 2018 (Fig. 1). The outbreak ward was screened 2–3 times a week during the outbreak period. Additional tests for VRE colonisation were performed during this period based on contact patients and wards closely related to the outbreak ward. From January 2019 onwards, patients admitted to the ward for at least ten days were tested every two weeks for VRE colonisation.

Additional infection prevention measures were taken during the VRE outbreak period. These include:

- Creating a cohort of positive patients and patients at risk of VRE. Nurses that work on the VRE-cohort do not work on the non VRE-cohort. Additional HCWs were used for night shifts to prevent nurses from working on both cohorts.
- More attention was given to hand hygiene measures. However, no measurements on compliance rates were performed during the outbreak.
- Additional use protective equipment like gloves and aprons were used for all patients.
- Intensified screening on VRE. Patients in the outbreak ward were screened three times per week. Admission screening and discharge screening for all patients.
- During a short period during the outbreak, the admission of new patients was completely halted.
- Contact tracing and screening of discharged patients, including screening at home.
- Intensified cleaning and even closure of a ward and moving patient to a secondary ward to clean the original ward.
- Environmental screening for VRE.

Between July–December 2018, 141 positive VRE tests were reported, with a peak of 25 positive tests in one week. In total, 48 patients tested positive for VRE over the study period.

### Modelling

The binary outcome variable Y was defined (1) and calculated using the VRE screening data over the study period.

1 $$Y= \left\{\begin{array}{cc}1,& \text{number of patients colonised with VRE in ward}>0\\ 0,& \text{otherwise}\end{array}\right.$$

We estimated the conditional probability that there is at least one patient colonised with VRE in a specific ward (Y) given the covariates pat_num, pat_num_ant, PR_pat_num and PR_pat_num_ant (2).

2 $$P\left(Y=1\right| pat\_num,pat\_num\_ant,PR\_pat\_num,PM\_pat\_num\_ant)$$

### Decision trees

A decision tree was used to determine a simple set of rules based on the covariates to estimate the conditional probability of Y [19]. The decision tree was grown using a 70% random training sample of the complete set of data. The data were split incrementally by adding question nodes. The question nodes consider the ability of each covariate to discriminate between the observed binary outcomes and formulates the question using the one that can discriminate best [20]. We used the Gini index to quantify the discriminatory ability of each covariate at the question nodes [19]. Continuing in this way, a tree branch structure is created, leading to the final decision or leaves of the tree.

### Random forest

The model performance of decision trees was improved by creating an ensemble of decision trees and using them in unison to predict the outcome variable [20]. We used the same 70% randomly sampled training samples used to train the decision tree model. To build the random forest (RF) model, 500 random samples with replacement (bootstrap sample) were drawn from the training data and two random outcome variables were used to build a decision tree for each of the bootstrap sample. The probability of Y was determined by calculating the proportion of the 500 trees that predicted $Y=1$.

We compared the model performance of the decision tree and random forest models using the remaining 30% data as a test sample. The area under the receiver operating characteristic curve (ROC) was used to measure model performance as it provides a holistic view of how well the model predicts the outcome variable for different levels of sensitivity and specificity [21]. An AUC between 0.7 and 0.8 is considered as acceptable and between 0.8 and 0.9 excellent [123].

### Software

The R statistical programming language was used to perform the analyses in this study [22]. Graphs were created and evaluated using igraph [23]. The decision trees and random forest models were built using the rpart and randomForest packages [24, 25]. In addition, the tidyverse R package was used to clean and structure the data [26].

## Results

In total, 48 distinct wards were occupied over the 730 days in the study period (2018–2019). Of the possible 35,040 observations, if all the wards were occupied every day, only 31,649 observations were collected, of which 1377 (5.45%) had at least one patient with VRE.

### Covariates

**Fig. 2:**
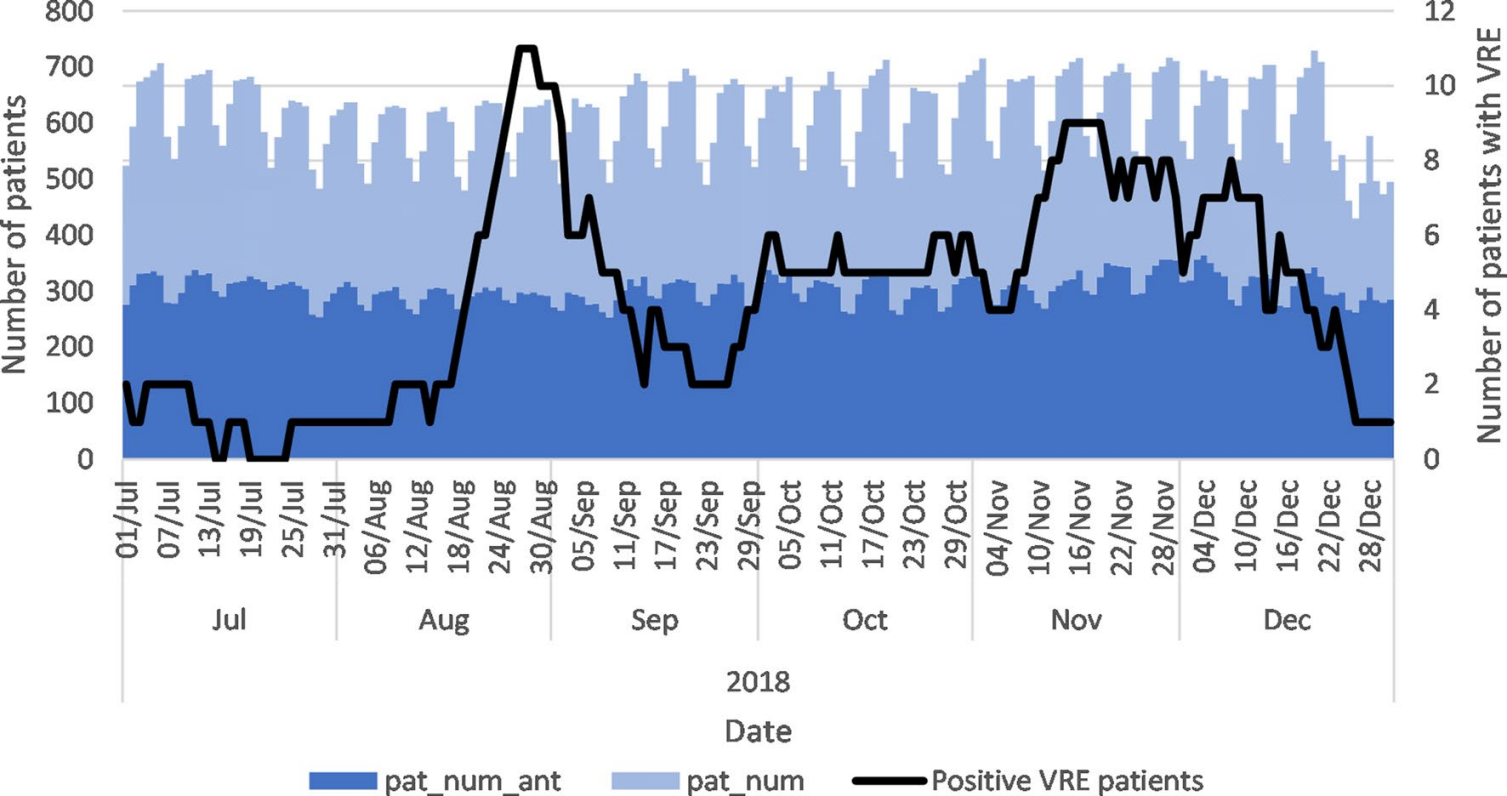
Number of patient and patients using antibiotics. pat_num_ant = the number of patients using antibiotics in each ward; pat_num = the number of patients in each ward

**Fig. 3:**
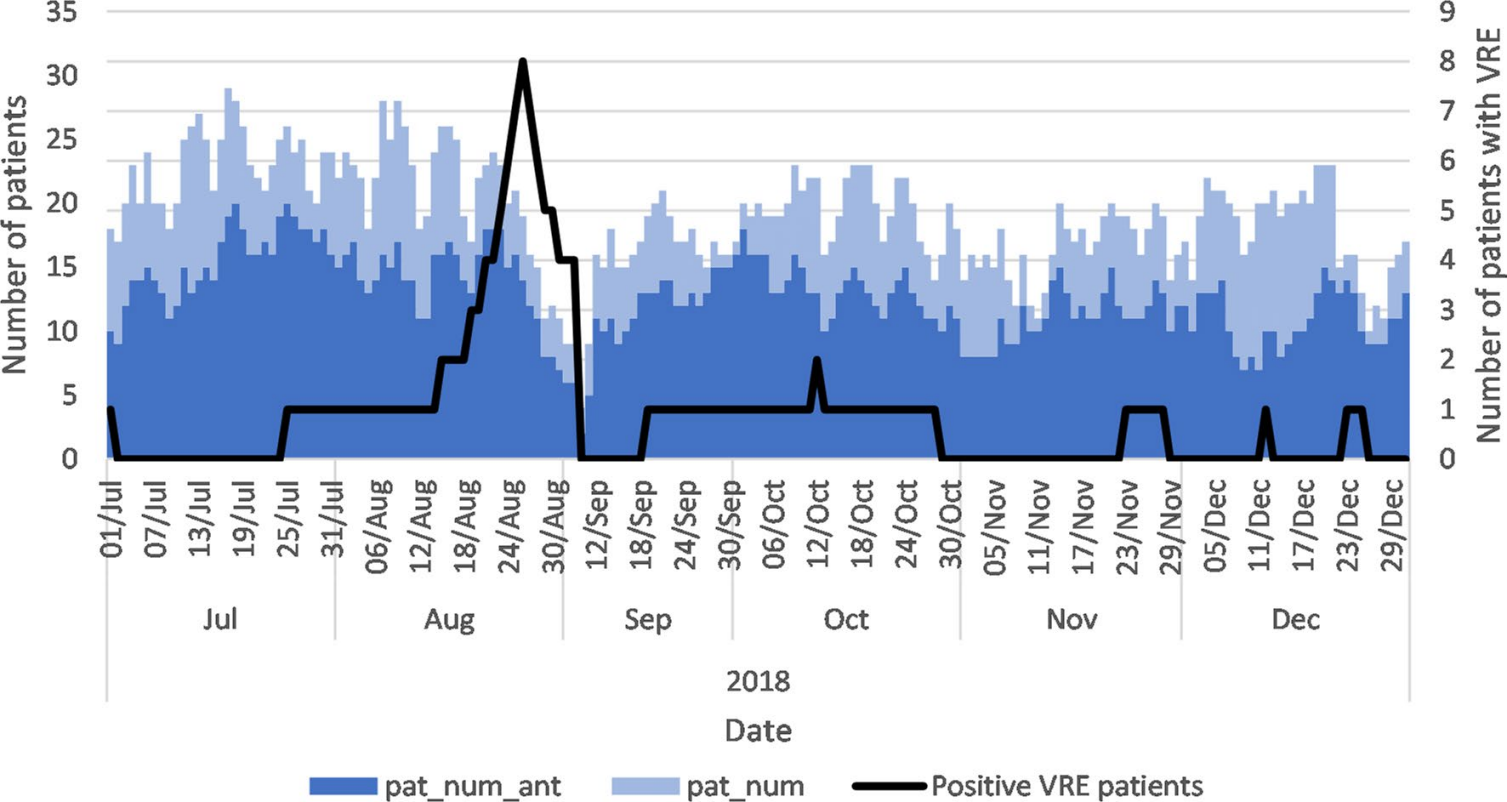
Number of patient and patients using antibiotics in example general care ward. pat_num_ant = the number of patients using antibiotics in each ward; pat_num = the number of patients in each ward

The pat_num and pat_num_ant covariates are shown with the number of patients colonised with VRE during the VRE breakout period in 2018 in Fig. 2. We highlight the general care ward with many VRE colonised patients during this outbreak in Fig. 3. These results show a higher level of variation at the ward level, which conforms better to the number of patients colonised with VRE. The highest number of VRE colonised patients were observed in the last week of August 2018. At the hospital level, the relationship between the pat_num_ant, pat_num and the number of VRE colonised patients is not evident. When the same data are shown at the ward level for the general care ward, these covariates are correlated with the number of VRE colonised patients.

**Fig. 4:**
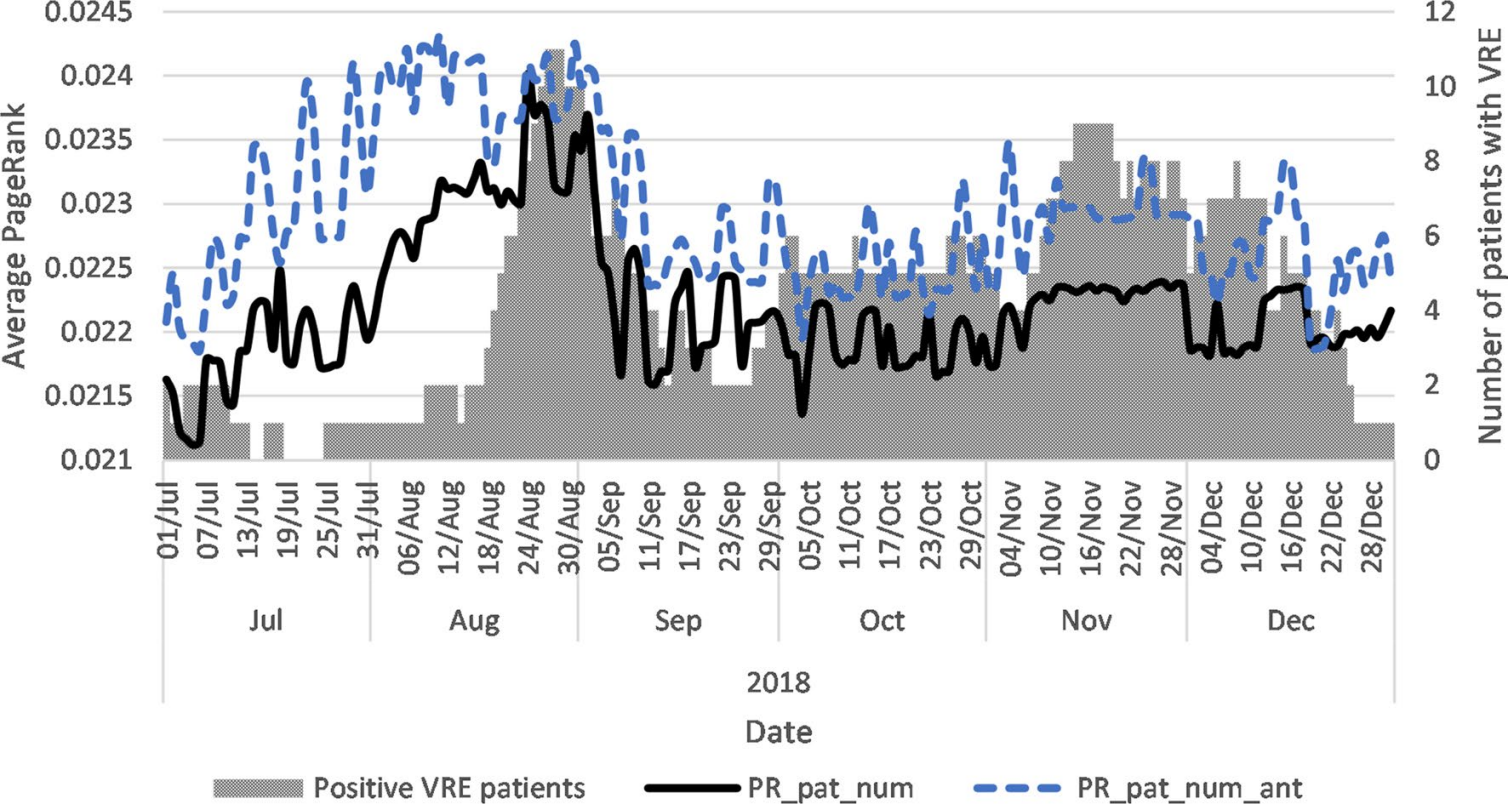
Average daily PageRank covariate and the number of VRE positive patients. PR_pat_num = PageRank of patient movements between wards; PR_pat_num_ant = PageRank of patient movements using antibiotics

**Fig. 5:**
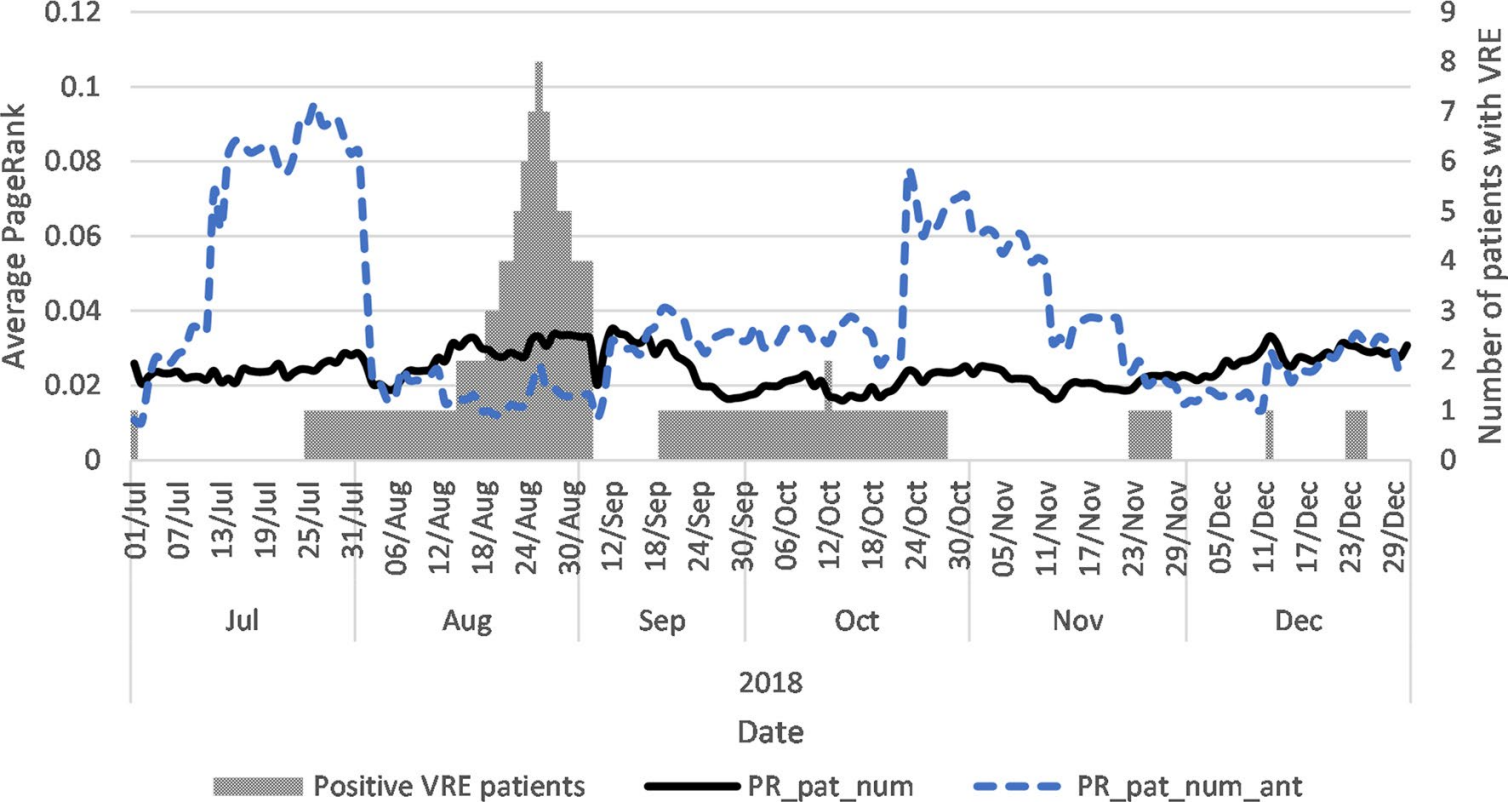
Average daily PageRank covariate and the number of VRE positive patients in example ward general care ward. PR_pat_num = PageRank of patient movements between wards; PR_pat_num_ant = PageRank of patient movements using antibiotics

Comparing the two PR_pat_num and PR_pat_num_ant reveal that during this period, PR_pat_num_ant was higher than PR_pat_num (Fig. 4). This means that, on average, the probability of a patient using antibiotics to visit a ward was higher than for the total patient population. The same covariates are shown for the example general care ward in Fig. 5. The general care ward experienced a significant increase in PR_pat_num_ant during July and October 2018, which lasted for four weeks and yet PR_pat_num did not show a similar pattern. These results show that the two centrality covariates provide different information of the patient and antibiotics flow in a hospital at the ward level.

### Decision tree

**Fig. 6:**
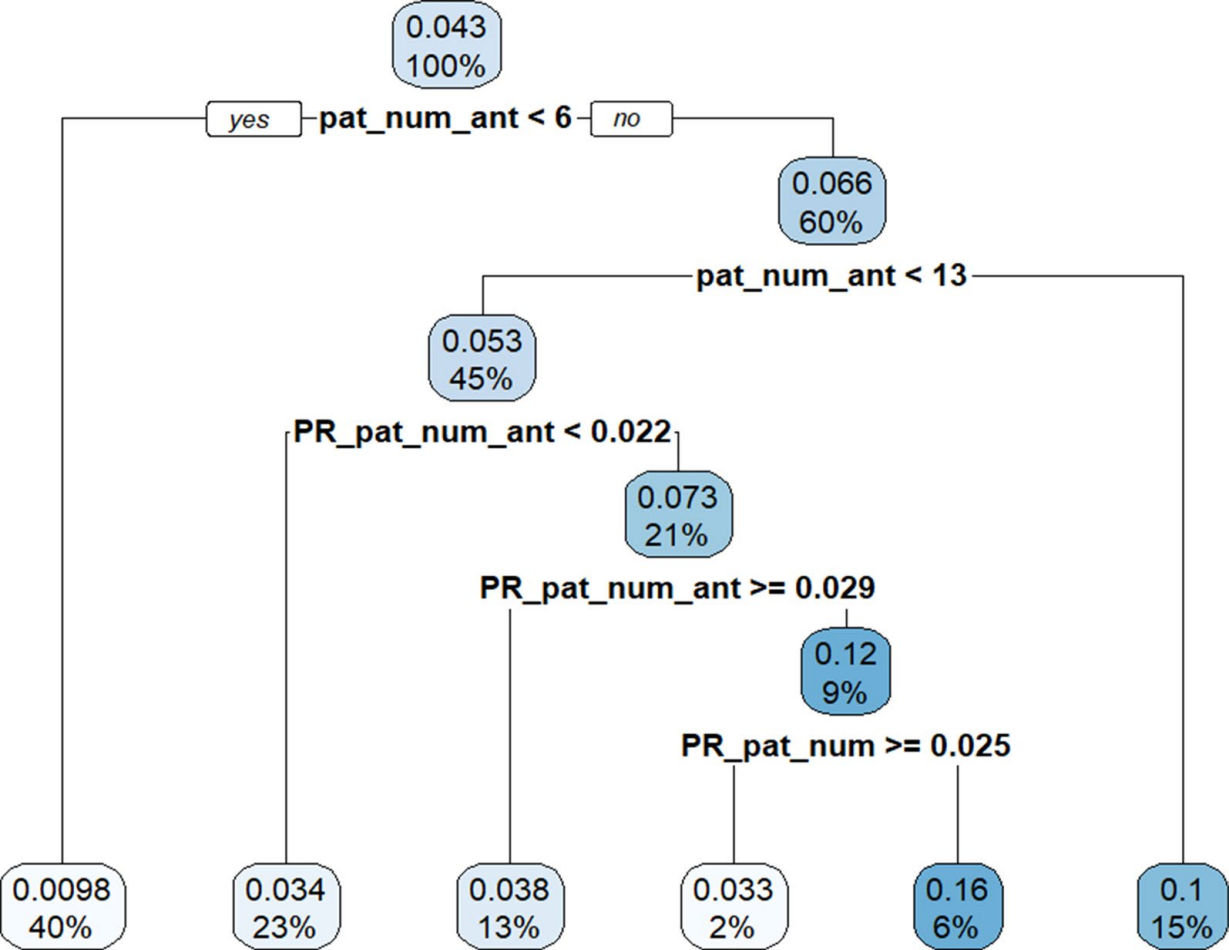
Decision tree for the daily VRE colonisation in a hospital ward using PageRank and traditional covariates. pat_num_ant = the number of patients using antibiotics in each ward; PR_pat_num_ant = PageRank of patient movements currently using antibiotics; PR_pat_num = PageRank of patient movements between wards. In each node, the percentage of wards with at least one patient colonised with VRE is shown above the sample distribution of the node

The 70% training sample had a 4.3% observations for which $Y=1$ at the root node (Fig. 6). The pat_num_ant covariate splits the first nodes. If the number of patients is less than six, which is the case for 40% of the training sample, then there is a 0.098% probability that the ward has a VRE colonised patient. If the number of patients in a ward is six or more, but less than 13, we continue to the next node to consider the PT_pat_num_ant covariate. After dividing the training sample by the five nodes, we arrive at the seven leaves of the tree. The probabilities of the leave population range between 0.98% and 15.68%. According to the order in which the covariates were used in the model, the pat_num_ant is the most important covariate to estimate the probability of a hospital ward having at least one patient colonised with VRE. The PR covariates are next in the order of importance to determine the final leaves of the tree. The decision tree results can be written and executed as a simple set of rules provided in (3).

3 $$P\left(Y=1\right| \mathrm{pat}\_\mathrm{num},\mathrm{pat}\_\mathrm{num}\_\mathrm{ant},\mathrm{PR}\_\mathrm{pat}\_\mathrm{num},\mathrm{PM}\_\mathrm{pat}\_\mathrm{num}\_\mathrm{ant})=$$

$$0.0098$$ if $$\mathrm{pat}\_\mathrm{num}\_\mathrm{ant}$$ $$<6$$,

$0.0326$ if $\mathrm{pat}\_\mathrm{num}\_\mathrm{ant}$ $\in [\mathrm{6,13}]$ AND $\mathrm{PR}\_\mathrm{pat}\_\mathrm{num}\_\mathrm{ant}$ $\in [0.022, 0.029)$ AND $\mathrm{PR}\_\mathrm{pat}\_\mathrm{num}$ $\ge 0.025$,

$$0.0340$$ if $$\mathrm{pat}\_\mathrm{num}\_\mathrm{ant}$$ $$\in [\mathrm{6,13}]$$ AND $$\mathrm{PR}\_\mathrm{pat}\_\mathrm{num}\_\mathrm{ant}$$ $$<0.22$$,

$$0.0384$$ if $$\mathrm{pat}\_\mathrm{num}\_\mathrm{ant}$$ $$\in [\mathrm{6,13}]$$ AND $$\mathrm{PR}\_\mathrm{pat}\_\mathrm{num}\_\mathrm{ant }\ge 0.29,$$

$$0.1030$$ if $$\mathrm{pat}\_\mathrm{num}\_\mathrm{ant}$$ $$\ge 13$$,

$0.1568$ if $\mathrm{pat}\_\mathrm{num}\_\mathrm{ant}$ $\in [\mathrm{6,13}]$ AND $\mathrm{PR}\_\mathrm{pat}\_\mathrm{num}\_\mathrm{ant}$ $\in [0.022, 0.029)$ AND $\mathrm{PR}\_\mathrm{pat}\_\mathrm{num}$ $<0.025$

### Random forest

**Fig. 7:**
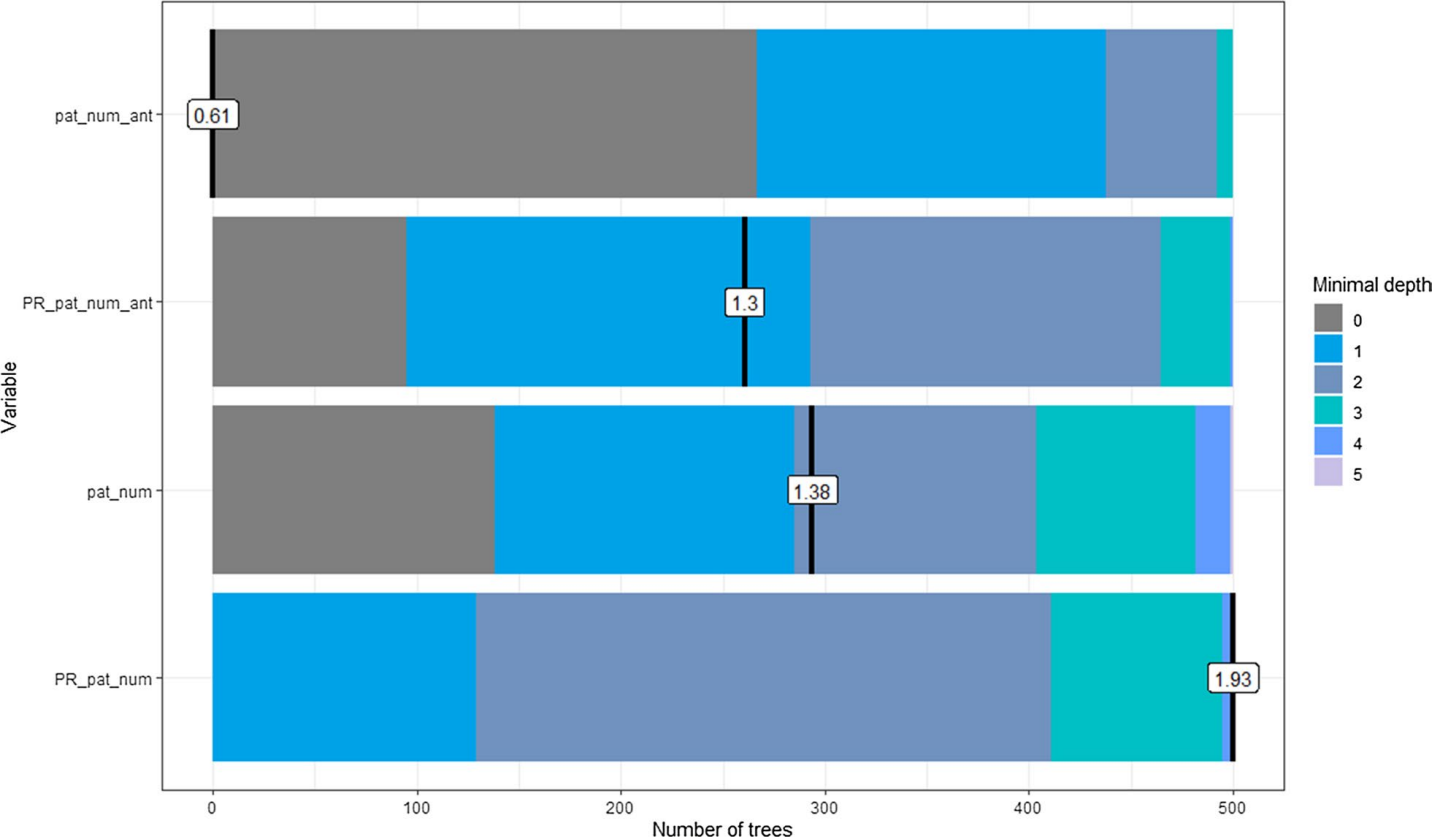
Minimal depth for each covariate in the 500 random forest decision trees. pat_num_ant = the number of patients using antibiotics in each ward; PR_pat_num_ant = PageRank of patient movements currently using antibiotics; pat_num = the number of patients in each ward; PR_pat_num = PageRank of patient movements between wards

The minimal depth provides insight into where a covariate occurs for the first time in the decision trees for the random forest and quantified variable importance. Covariates with lower minimal average depth are used to split larger proportions of the population due to higher discriminatory power. The results show that pat_num_ant has the lowest average depth (0.61) and is most likely to be used in the root node. This result is consistent with our single decision tree model (Fig. 7). PR_pat_num was not used as a root node for any of the 500 decision trees. It has the largest average depth (1.93) in the trees, which means that it was generally used in nodes appearing lower in the decision trees.

**Fig. 8:**
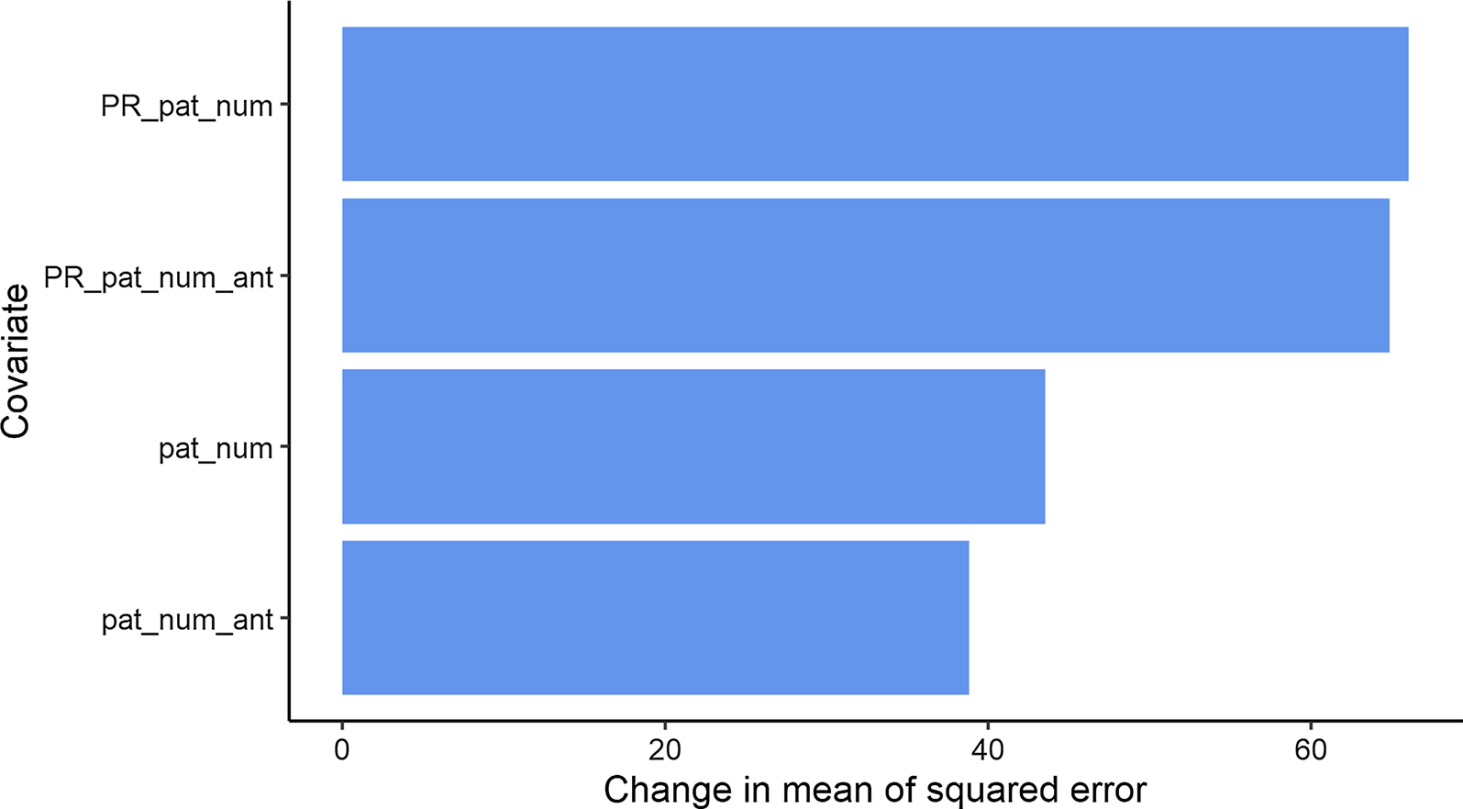
The change in mean squared error when covariate values are replaced with random values. PR_pat_num = PageRank of patient movements between wards; PR_pat_num_ant = PageRank of patient movements currently using antibiotics; pat_num = the number of patients in each ward; pat_num_ant = the number of patients using antibiotics in each ward

**Fig. 9:**
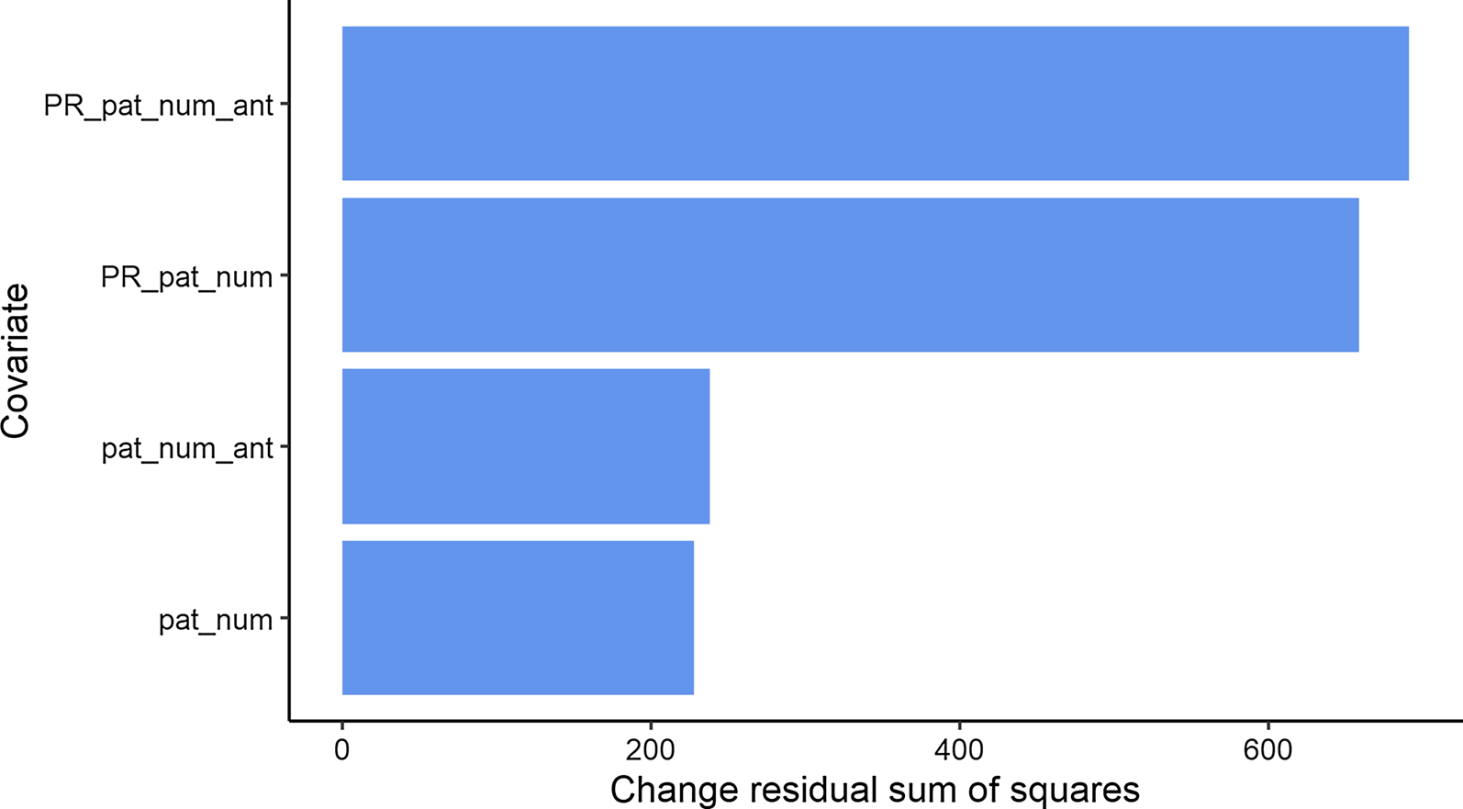
The change in residual sum of squares when covariate values are replaced with random values. PR_pat_num_ant = PageRank of patient movements currently using antibiotics; PR_pat_num = PageRank of patient movements between wards; pat_num_ant = the number of patients using antibiotics in each ward; pat_num = the number of patients in each ward

We determined the covariate importance in the RF model by calculating the percentage increase in the mean square error (MSE) and the change in the residual sum of squares (RSS) of the model should random information replace the values of the model covariates. The results show that the PR covariates are the most important ones in terms of the MSE (Fig. 8) and RSS (Fig. 9) reductions.

### Model performance

**Fig. 10:**
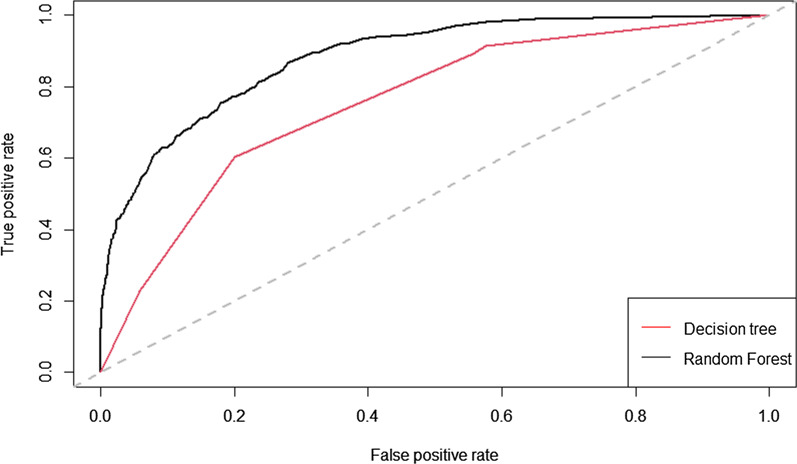
Lorenz curves of the decision tree and random forest models

The performance of the models is compared to the Lorenz curves shown in Fig. 10. The Lorenz curve of the RF model is consistently higher than for the decision tree model. The RF model achieved an area under the curve of 0.883 and the decision tree model 0.755 on the 30% test set. This result confirms that the random forest model performs better than a single decision tree for all levels of model sensitivity and specificity on data not used to estimate the models. This is important to estimate the loss in model performance when choosing to use the simple set of rules produced by the decision tree model to calculate the probability of Y rather than using the RF model.

## Discussion

This study showed how the movements of patients inside hospitals and their use of antibiotics could predict the VRE colonisation of patients at the hospital ward level. Two daily centrality measures were proposed to summarise the flow of patients and antibiotics at the ward level. A simple set of rules were produced which can be used to monitor the risk of VRE colonisation in hospital wards. Using an ensemble method, a more accurate but more complicated model was developed, which can be applied to the same effect should resources allow for it.

The two PageRank covariates proposed offered new insight into the centrality of wards regarding patient and antibiotic movements and their interaction. This study used the covariates to predict VRE colonisation, but they can be used in many other studies concerning antimicrobial resistance in hospitals. Institutional surveillance monitors the usage of antibiotics but not the flow and concentration thereof. The proposed PR covariates can be used in conjunction with existing institutional surveillance metrics to monitor the risks for VRE colonisation and AMR colonisation in general.

The decision tree model resulted in six simple questions and provided the probability that a ward has at least one patient colonised with VRE as an answer. This model enables hospitals to use passive data collected in their electronic health records to calculate this probability. To improve the accuracy of this model, a random forest model was built, which outperforms the decision tree model. The random forest model results were not as easily interpretable as that of the decision tree as it uses 500 smaller decision trees every time a probability is calculated. In practice, the model used will depend on the skills and resources of the hospital and its infection prevention and control specialists.

### Future work

The results of this study can be used to develop an early warning system for VRE colonisation and other microorganisms with similar transmission mechanisms. The probabilities produced by the models presented can be used to classify the predicted VRE colonisation outcome according to the desired level of sensitivity and specificity for such a system. The results can then be updated daily or as frequently as the covariates can be calculated and evaluated by the infection prevention specialists to decide on the best course of action.

Our results showed that the value of the patient movement and antibiotic PR covariates sometimes move in the opposite direction over time. This divergence suggests that the proportion of patients using antibiotics changes over time. These covariates can be used together to determine if emerging divergences increase the risk of VRE colonisation.

### Limitation

The study period was limited by the amount of data available for intrahospital patient movement, antibiotic use and VRE colonisation screening. UMCG migrated to a new electronic healthcare system in 2017, resulting in the antibiotic data not being available at the time of publication. There was a VRE outbreak in 2017, which would have allowed us to build these models on the 2017 outbreak and validate them on the 2018 outbreak. Once these data become available, this could be a future research opportunity.

Even though this study can determine if a patient were using antibiotics at a particular time, we could not distinguish between the types of antibiotics used. Some antibiotics target specific bacteria and can have a more significant effect on the risk of being colonised with VRE. A future research opportunity is to create antibiotics centrality measure for antibiotics targeting different bacteria.

The risk of VRE colonisation may differ between the types of hospital wards. For instance, patients admitted to an ICU may be more likely to be colonised with VRE than for general wards. We assumed that the difference in risk of VRE colonisation at the ward level could be explained by the number of patients, their antibiotics usage, and how patients in general and patients using antibiotics transition towards each ward using the centrality measures proposed in this study. For instance, the probability that at least one patient is colonised with VRE for a ward having a large number of patients using antibiotics and a relatively high probability that a patient using antibiotics may end up there compared to a general ward with low antibiotics usage. Even though the proposed models could predict VRE colonisation at the ward level, they may be further expanded to the patient level to test this assumption. This expansion will require additional patient data regarding demographics and comorbidities affecting the risk of VRE colonisation. A prediction model for VRE colonisation at the patient level using the proposed spatiotemporal centrality measures and patient-level data will also improve the efficiency of infection prevention specialists to control AMR in hospitals.

Even though the models focussed on predicting VRE colonisation at the ward level, the proposed spatiotemporal centrality measures may be used to generalise the models for other transmittable microbes in hospital environments. Future research may validate the relevance of these measures in a multicentre study using other outcome variables based upon, for example, carbapenem-resistant *Enterobacteriaceae* (CRE) or *Clostridium difficile*.

## Conclusion

This study showed how the movements of patients inside hospitals and their use of antibiotics could predict the VRE colonisation of patients at the ward level. Two daily centrality measures were proposed to summarise the flow of patients and antibiotics at the ward level. A simple set of rules was produced which can be used to monitor the risk of VRE colonisation in hospital wards. A random forest ensemble model was compared with a decision tree model to improve the prediction performance at the cost of simplicity. An early warning system for VRE colonisation can be developed to test and further develop infection prevention plans and outbreak strategies using these results.

## Data Availability

The data that support the findings of this study are available from UMCG but restrictions apply to the availability of these data, which were used under license for the current study, and so are not publicly available. Data are however available from the authors upon reasonable request and with permission of UMCG.

## Abbreviations

AMR: Antimicrobial resistance
AUC: Area under the curve
CI: Confidence interval
CRE: Carbapenem-resistant Enterobacteriaceae
DG: Dynamic directed spatiotemporal graph
EHR: Electronic health record
HCW: Healthcare worker
ICU: Intensive care unit
MSE: Mean square error
NGS: Next generation sequencing
PCR: Polymerase chain reaction
PR: PageRank
RF: Random forest
ROC: Receiver operator characteristic
RSS: Residual sum of squares
UMCG: University Medical Center Groningen
VRE: Vancomycin-resistant Enterococcus
